# Outcomes of patients receiving non-invasive ventilation (NIV) in the general intensive care unit: is NIV duration an important factor?

**DOI:** 10.1186/2197-425X-3-S1-A174

**Published:** 2015-10-01

**Authors:** L Wilson, J Gross, G Gallagher, A Wolff

**Affiliations:** Royal Free London NHS Foundation Trust, Intensive Care Unit, Barnet Hospital, London, United Kingdom

## Introduction

The role of non-invasive ventilation in the intensive care unit (ICU) is generally poorly understood. Published guidelines largely relate to the use of NIV outside the ICU^1,2^. Whilst much focus has been placed on relating indication for NIV on outcome, little attention has been paid to the duration of NIV and whether or not this might have an impact on outcome.

## Objectives

To review the practice and outcomes of NIV in our institution over a 1-year period and compare those receiving NIV for shorter ( < 48 hours) compared with longer (>48 hours) periods.

## Methods

We conducted a retrospective care note analysis of all patients receiving their first NIV period at any time during their ICU admission between 1^st^ January 2014 and 31^st^ December 2014. Patients receiving NIV after a period of mechanical ventilation were excluded from this analysis. Patients were analysed depending on whether they received NIV for a total period of < 48 hours or > 48 hours. For each group, data was collected for % time NIV actually received during their NIV period, number requiring intubation and mechanical ventilation, ICU length of stay and number surviving to ICU discharge.

## Results

**Figure 1:**
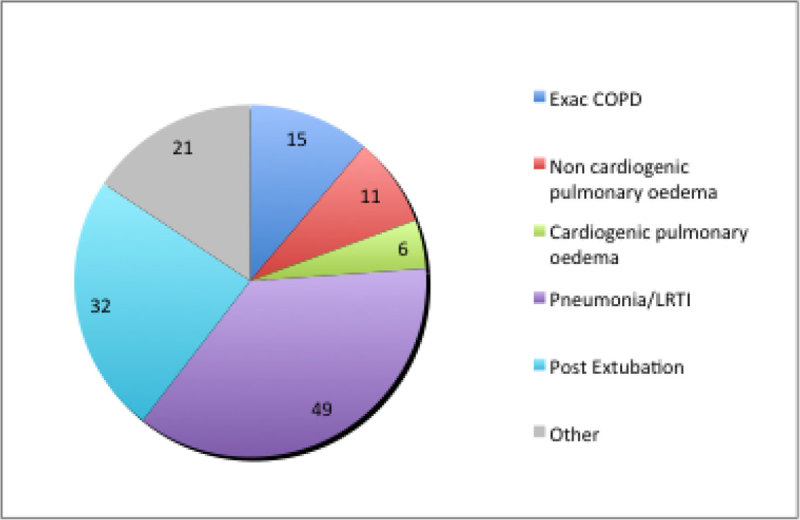
**Indications for NIV.**

The notes of 103 patients were reviewed. 49 were female (48%) with a mean age of 69 years (range 19-100 years). Indications for initiation of NIV are highlighted in figure 1.

Sixty-nine (67%) patients received NIV for < 48 hours compared with 34 (33%) patients receiving NIV for > 48 hours. Predicted acute hospital mortality (according to ICNARC score) on ICU admission was similar between the 2 groups (median predicted mortality risk 36% [IQR18.5-62.5] v 46% [IQR 15-68]; p = 0.34). When comparing those who received NIV for < 48 hours with those who received NIV for > 48 hours, the median total intermittent duration of NIV was 12 [IQR 5-18] and 136 [IQR 72-227] hours respectively (p < 0.05) and the median % of time actually spent receiving NIV during these periods were 100% [IQR 91-100] and 37% [IQR 18-71] respectively (p < 0.05). The median ICU length of stay was significantly shorter in the < 48 hour duration group (4.9 [IQR 3-11] days v 11.3 [IQR 7-23] days (p < 0.05)) although intubation rates (33% v 47%; p = 0.18) and % surviving to ICU discharge (70% v 74%; p = 0.68) were similar and not significantly different (table 1).

**Table 1:** Comparing outcomes according to duration of NIV.

	NIV for < 48 Hours	NIV for > 48 Hours	P-Value
**Number of patients**	69	34	
**Median % predicted acute mortality (ICNARC) [IQR]**	36 [18.5-62.5]	48 [15-68]	0.34
**Median time period of initial NIV administration (hours) [IQR]**	12 [5-18]	136 [72-227]	< 0.05
**Median % time NIV actually received during 1st period of administration [IQR]**	100 [91-100]	37 [18-71]	< 0.05
**Number intubated**	23 (33%)	16 (47%)	0.18
**Median time for mechanical ventilation for those intubated (days) [IQR]**	9 [5-10.5]	9 [5.5-19.5]	0.36
**Median ICU length of stay (days) [IQR]**	4.9 [3-11]	11.3 [7-23]	< 0.05
**Number surviving to ICU discharge**	48 (70%)	25 (74%)	0.68

## Conclusions

At our institution, those receiving NIV for < 48 hour time period had a shorter length of ICU stay compared with those receiving NIV for longer periods. Duration of NIV may be an important factor in determining success of NIV and may be worth further exploration in future prospective studies.
